# Portable infrared imaging for longitudinal limb volume monitoring in patients with lymphatic filariasis

**DOI:** 10.1371/journal.pntd.0007762

**Published:** 2019-10-04

**Authors:** Celia Zhou, Channa Yahathugoda, Lalindi De Silva, Upeksha Rathnapala, Grant Owen, Mirani Weerasooriya, Ramakrishna U. Rao, Gary J. Weil, Philip J. Budge

**Affiliations:** 1 Department of Biochemistry and Molecular Biology, Wake Forest University, Winston-Salem, North Carolina, United States of America; 2 Summer Research Program, Institute of Public Health, Washington University School of Medicine, St. Louis, Missouri, United States of America; 3 Filariasis Research Training and Services Unit, Faculty of Medicine, University of Ruhuna, Galle, Sri Lanka; 4 Infectious Diseases Division, Department of Medicine, Washington University School of Medicine, St. Louis, Missouri, United States of America; Hitit University, Faculty of Medicine, TURKEY

## Abstract

**Background:**

The Global Programme to Eliminate Lymphatic Filariasis (LF) emphasizes hygiene, exercise, and other measures to reduce morbidity and disability related to LF. We recently reported that a portable, three-dimensional, infrared imaging system (3DIS) provides accurate limb volume measurements in patients with filarial lymphedema. To assess the practical utility of repeated 3DIS measurements for longitudinal lymphedema management, we examined intraday and day-to-day leg volume changes in adults with filarial lymphedema in southern Sri Lanka.

**Methodology and principal findings:**

We assessed 41 participants with lower extremity lymphedema (stages 1–6) in their homes in the mornings (6:00–9:00 AM) and afternoons (2:00–6:00 PM) of three days within one calendar week. Two examiners performed replicate 3DIS volume measurements at each visit. Median coefficient of variation among replicate volume measurements was 1.7% (IQR 1.1% - 2.3%) for left legs and 2.2% (IQR 1.6% - 2.8%) for right legs. Median intraday volume increase was 3.0%. Range among daily volume measurements tended to be lower for afternoon measurements (median 2.25%, IQR 1.4%– 5.4%) than for morning measurements (median 3.0%, IQR 1.4% - 8.4%).

**Conclusions and significance:**

Limb volume measurements by 3DIS are accurate and reproducible, and this technique is feasible for use in patients’ homes. We have developed practical suggestions for optimal outcomes with 3DIS. Duplicate measurements should be performed and repeat assessments should be done at approximately the same time of day to minimize bias. Duplicate measures that vary by more than 8% should prompt review of scanning technique with a repeat measurement. With proper training and attention to technique, 3DIS can be a valuable tool for healthcare workers who work with lymphedema patients.

## Author summary

Morbidity Management and Disability Prevention (MMDP) is a critical but neglected component of the Global Programme to Eliminate Lymphatic Filariasis. Lack of infrastructure, trained practitioners, political will, and resources are challenges to effective implementation of MMDP services. This is compounded by the paucity of treatment options for limb lymphedema. Currently daily washing, elevation, and exercise are the only therapy available to most patients with filarial limb lymphedema, and new treatments are sorely needed. We previously reported that portable three-dimensional infrared imaging provides rapid, accurate, and reproducible limb measurements in patients with filarial lymphedema. Here, we show that similarly accurate and reproducible limb measurements are possible outside the clinic (in or outside patients’ homes). Using this technique we quantify how much limb volume varies from day to day and during the day for patients with filarial lymphedema, and provide recommendations for the optimal use of this tool in longitudinal monitoring of limb volume in research or in the clinic.

## Introduction

Lymphatic filariasis (LF) is a tropical, mosquito-borne, parasitic infection that causes lymphatic dysfunction leading to limb and genital lymphedema in tens of millions of affected individuals worldwide. The World Health Organization’s Global Programme to Eliminate Lymphatic Filariasis (GPELF) has prevented over 80 million new LF cases via systematic distribution of preventive chemotherapy in many endemic nations [1, 2]. Unfortunately, millions already affected by filarial lymphedema and those who will yet become affected before global elimination of LF transmission is achieved will require lifelong, active daily management of their lymphedema to prevent progressive worsening. Like all conditions requiring daily care, the ability to monitor improvement or worsening over time provides vital feedback to both patients and clinicians. The ability to monitor lymphedema progression is also essential in clinical trials for new or improved lymphedema therapies, which are sorely needed.

Studies of filarial lymphedema often use the clinical staging system of Dreyer et al [3] to monitor lymphedema progression [3–6]. This system is a very useful way to categorize lymphedema severity but it has some drawbacks as an outcome in clinical trials. It is an ordinal (not interval) scale and therefore lacks sensitivity in detecting potentially meaningful changes that do not result in progression (or regression) of stage. It also requires the examiner to make judgments that may vary from observer to observer, which can hinder reproducibility. Finally, it does not necessarily correlate with progression of disability, which may be a more meaningful outcome to the afflicted patient. For these reasons, interval outcome measures such as limb size, skin thickness or impedance provide important complementary data to clinical staging [7].

Limb volume measurement is another way of monitoring lymphedema progression that is both continuous and highly relevant to disability experienced by lymphedema patients. Historically, water displacement was the gold standard for measuring limb volumes, but it is logistically difficult to implement and infrequently used. We recently reported that a portable three-dimensional infrared imaging system (3DIS) enabled rapid and accurate lower extremity volume measurements comparable to those obtained by water displacement while requiring much less time and with less inconvenience to patients [8]. To understand the utility of repeated 3DIS measurements of limb volumes, however, one needs to understand both the amount of variability in the method, and how much limb volumes may actually vary in the absence of true lymphedema progression. Thus, the objectives of this study were to determine: 1) whether 3DIS is capable of detecting intraday and day-to-day variations in limb volume among persons with filarial lymphedema, and 2) describe the magnitude of these changes (if detectable). Because our study design required each patient to be examined many times in the course of a week, a third objective was to assess the practicality of doing 3DIS measurements in the community.

## Methods

### Ethics statement

The institutional review board at Washington University School of Medicine in St. Louis and the ethics review committee at the Faculty of Medicine, University of Ruhuna, Galle, Sri Lanka, approved the study protocol. Participants received information sheets describing the study in English and Sinhala (native language) and provided written informed consent to participate in the study.

### Population and setting

We recruited participants over eighteen years of age who previously or currently receive lymphedema care at the Filariasis Research Training and Services Unit (FRTSU) at the Faculty of Medicine, Galle, Sri Lanka. Inclusion criteria included the presence of lymphedema in one or both legs and the ability to stand up to 5 minutes with limited assistance. There are an estimated 1096 patients with filarial lymphedema in Galle district [9]. Among these, an estimated 8% have advanced lymphedema (stage 4 or higher). Care for patients with filarial lymphedema in Galle District is provided at FRTSU and at two filariasis clinics run by the national Anti-Filariasis Campaign [10].

We conducted the study in June 2018 with a target enrollment of sufficient participants to include at least ten legs of each lymphedema stage 0, 1, 2, 3, 5, and 6 (stage 4 limbs, which are characterized by nobs without deep folds or mossy lesions [3], are uncommon). Sample size was ultimately limited by logistical constraints—we visited as many patients as possible during the time in which the study team was available. The final study population was a convenience sample of subjects selected by stage and proximity of residence to other participants to allow the study team to visit as many participants as possible each day. We did not power to detect differences in volume changes by stage. Rather, we sought to study the ability of 3DIS to detect day-to-day and intraday volume changes. To make this characterization more robust, we included participants of all stages.

### Study procedures

**Fig 1:**
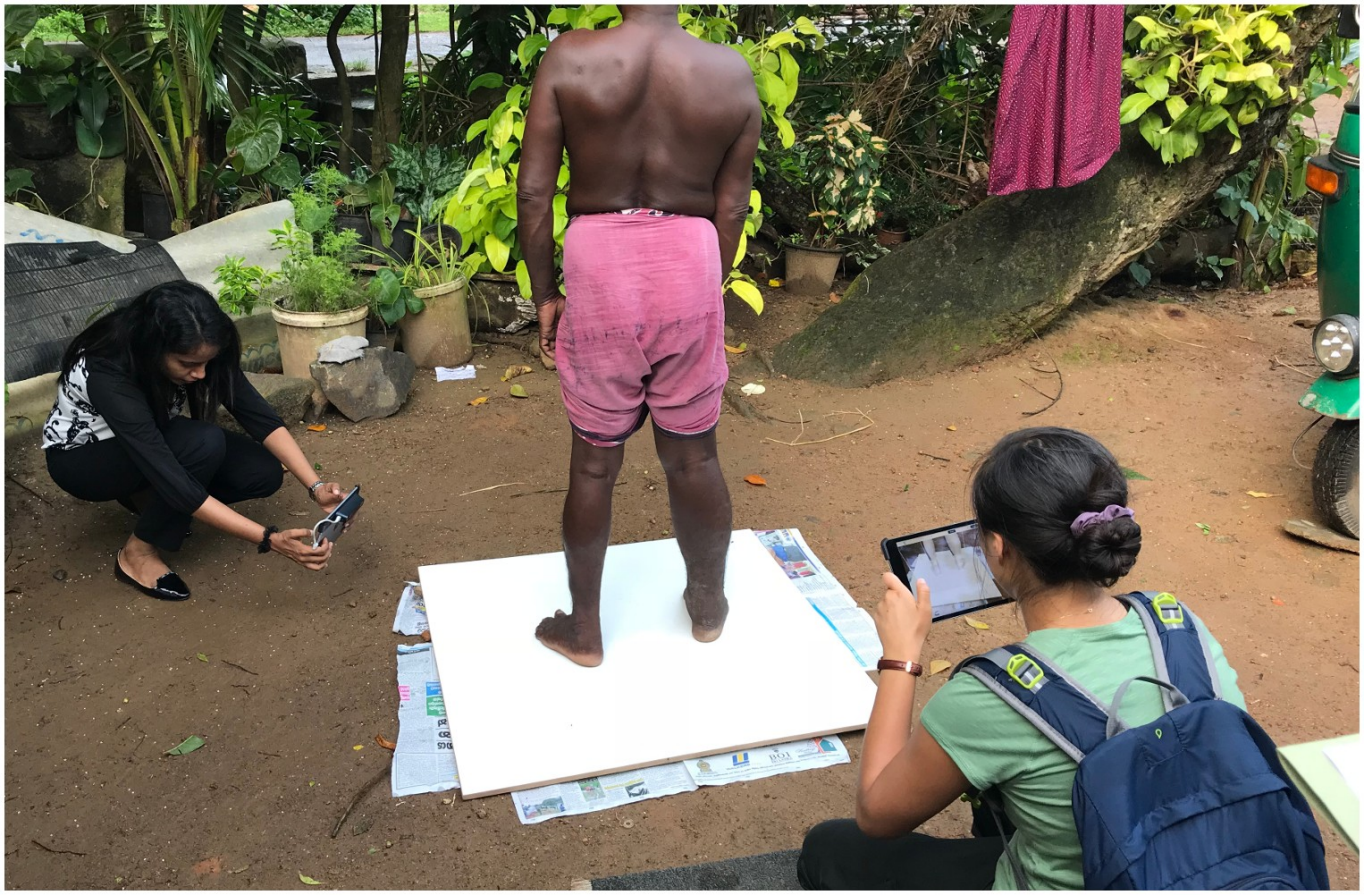
Study staff concurrently examining a participant with right leg lymphedema.

A study team consisting of two examiners and a scribe visited participants in their homes on three days within one calendar week. The three study days were not necessarily consecutive. We chose a short (one week) time frame in order to capture day-to-day variability in limb volumes in the absence of true lymphedema progression. The team visited between four and ten participants each study day in the morning (between 6:00–9:00 AM) and the afternoon (between 2:00–6:00 PM). At each visit both examiners used the 3DIS system (LymphaTech, Atlanta, Georgia) to collect 3D scans of both limbs of the participant as previously described [8]. Each scan requires that the participant stand on a flat, smooth surface in an open area with room for the examiner to circle (walk around) the participant at a distance of about 1 meter. In some homes, this required that the team (with the participant’s permission) rearrange furniture to create the necessary space. At homes where the indoor space was inadequate or the floor was uneven, the participant stood on a reinforced platform in a shaded area outdoors (Fig 1). The team tested all standing surfaces to ensure they were level (within 1° of horizontal in all directions) using a bubble level or the iPhone level app prior to scanning. Participants stood with legs bare from the knees down and their heels spaced approximately 25 cm apart (the distance between the heels was set using a plastic spacer to ensure a uniform stance), while the examiner circled the participant in a counterclockwise fashion to collect the 3D imaging data. To decrease the time participants were required to stand still, two examiners scanned concurrently at most visits (Fig 1).

The 3D infrared imaging system (LymphaTech, Atlanta, Georgia) consists of a portable infrared sensor (Structure by Occipital, San Francisco, California) mounted on a tablet computer. It employs proprietary software to combine depth data from the sensor with accelerometer data from the tablet to create point-cloud reconstructions of the surface of the scanned limbs. The LymphaTech software analyzes the 3D model to calculate customizable measurements of limb volume and circumferences. We chose to calculate leg volumes at a height of 32 cm, because this height was validated vs. water displacement in our prior study [8].

### Examiners

Examiners were trained individually by two of the authors with prior experience (PJB and CY) using a standard operating procedure (SOP) document. Trainers demonstrated the technique, then allowed the learner to practice the technique until their scans were reliably free from errors. Examples of common mistakes were demonstrated and a written troubleshooting guide for recognizing and avoiding potential errors was provided. The training process usually takes about an hour, but examiners continue to learn by experience with each scan, since the LymphaTech software shows the captured 3D model after each scan, which examiners are required to carefully review for errors before saving it to the database.

### Data capture

Acquiring volume measurements by 3DIS has two components: (1) acquisition of the point cloud model, and (2) analysis of the model to calculate measurements of the limb. After our initial validation of the 3DIS system [8] LymphaTech updated the software for both functions. We began the current study using an updated software package (LT-NTD) for both acquisition and analysis. Advantages of the new software were that it performs both acquisition and analysis on the iPad tablet, bypassing the need to upload the acquired point cloud model to a separate computer, and that it provides better resolution of the foot/floor border. However, during the course of the study we discovered that although acquisition of data using the new LT-NTD software was reliable, analysis of the 3D models using LT-NTD was not. We reported this to LymphaTech and they discovered that the manufacturer of the Structure infrared sensor (Occipital, San Franscisco, CA), a component of the 3DIS system, had made a software change responsible for the problem. We therefore analyzed all acquired 3D models with an older version of LymphaTech software (LT-V2). Additionally, a bug in the new software caused it to crash when the stored memory became too large, causing us to lose data from 12 study visits. After discovering these issues, examiners added “backup” scans using the older acquisition software. Thus, we collected 6 replicate scans per visit for the majority of visits: two primary scans (collected with LT-NTD) and one backup scan (collected with LT-V2) per examiner. Exceptions included visits to participants who had difficulty standing and could not tolerate a full complement of scans. Rarely, examiners acquired more than six scans per visit when the team was concerned about the quality of one or more of the initial scans.

### Data management and analysis

Both LT-NTD and LT-V2 export volume and circumference measurements directly to MS Excel. Data was imported to STATA version 12.1 (College Station, Texas) for all statistical analyses. At the time of data analysis we noted that3D models acquired with LT-NTD gave volume measurements consistently larger than scans acquired by LT-V2 at the same visit. LymphaTech indicated that this offset was due to the software change in the Structure sensor, for which LT-NTD had not been appropriately calibrated. This meant that we could not directly compare the LT-V2 and LT-NTD measurements without introducing systemic error. Rather than discard the data collected with LT-NTD, we elected to adjust for the systemic error. We first determined the magnitude of the error by linear regression of the LT-V2 and LT-NTD scans taken at the same visits. Volume measurements from the primary scans were log-transformed to achieve a normal distribution (tested by STATA’s “sktest” function), then regressed against similarly transformed volume measurements from the matching backup (LT-V2). This revealed a mean difference between primary and backup volume measurements of 5.4%, but the difference trended lower as limb volume increased (Fig 1A). Therefore, rather than apply a blanket adjustment of 5.4% to all LT-NTD measurements, we used the offset and slope from the linear regression to adjust the volume of each individual primary scan according the formula: adjusted volume = exp(ln((initial volume—850) x 1.050903)-0.5111012).

### Quality control and exclusion of scans

Improper scanning technique can cause distortions in the 3D models resulting in measurement errors. The primary safeguard against inclusion of poor quality scans is visual inspection of each 3D point cloud model at the time of acquisition. Examiners were trained to inspect each model carefully immediately after acquisition and to reject any models with distortions or incomplete capture. After spot checks revealed that some distorted scans nonetheless made their way into the database, we performed a secondary review of all scans and rejected 43 of 1256 scans (3.4%) due to distortions. Examples of these scans and the reasons for exclusion are provided in S1 Fig.

## Results

### Enrollment

Forty-one participants enrolled in the study; 33 (80%) were female. The median age of study participants was 56 years (range 35–73). Table 1 shows the lymphedema stage and volumes of the participants’ legs. Eighteen participants (44%) had unilateral lymphedema, 23 (56%) had bilateral lymphedema (i.e. both legs stage ≥ 1). Among those with bilateral lymphedema, ten had right and left legs of equal stage. To preserve independence of observations in our analyses, we therefore analyzed data from right and left limbs separately.

**Table 1:** Number and volume of participant limbs, by stage. Data shown represent the number of limbs of each stage (N), and the median, interquartile range (IQR) and range of volumes among left and right limbs of that stage. The mean morning volume (averaged across the three study days) for each limb is represented.

Leg	Stage	N	Volume (mL)		
			Median	IQR	Range
Left	0	10	1896	1789–2198	1513–2349
	1	6	2139	1947–2291	1896–2484
	2	10	2488	2126–2968	1506–3918
	3	8	2856	2445–3440	1899–3708
	5	3	3806	3461–4062	3461–4062
	6	4	5173	3505–7479	2350–9273
	Left total	41	2350	1947–3069	2350–9273
Right	0	8	1906	1567–1998	1443–2828
	1	6	2116	1881–2336	1784–2550
	2	8	2526	2322–2701	2130–2908
	3	8	2924	2637–3005	2299–3186
	5	5	3862	3471–4169	3430–4441
	6	6	3503	3431–5143	2411–5446
	Right total	41	2590	2130–3186	1443–5446

In total, examiners made 246 visits (six per participant) and acquired 1,256 3D images (scans). Data from one study day (12 visits, six participants) were lost due to an unanticipated software crash, leaving data from 232 visits and 1256 scans available for analysis. Visual review of all 3D image files identified 41 scans (3.2%) of poor quality that were excluded from analysis, leaving data from 1,213 scans in the final dataset (S1 Table).

### Reproducibility of replicate scans per visit

**Fig 2:**
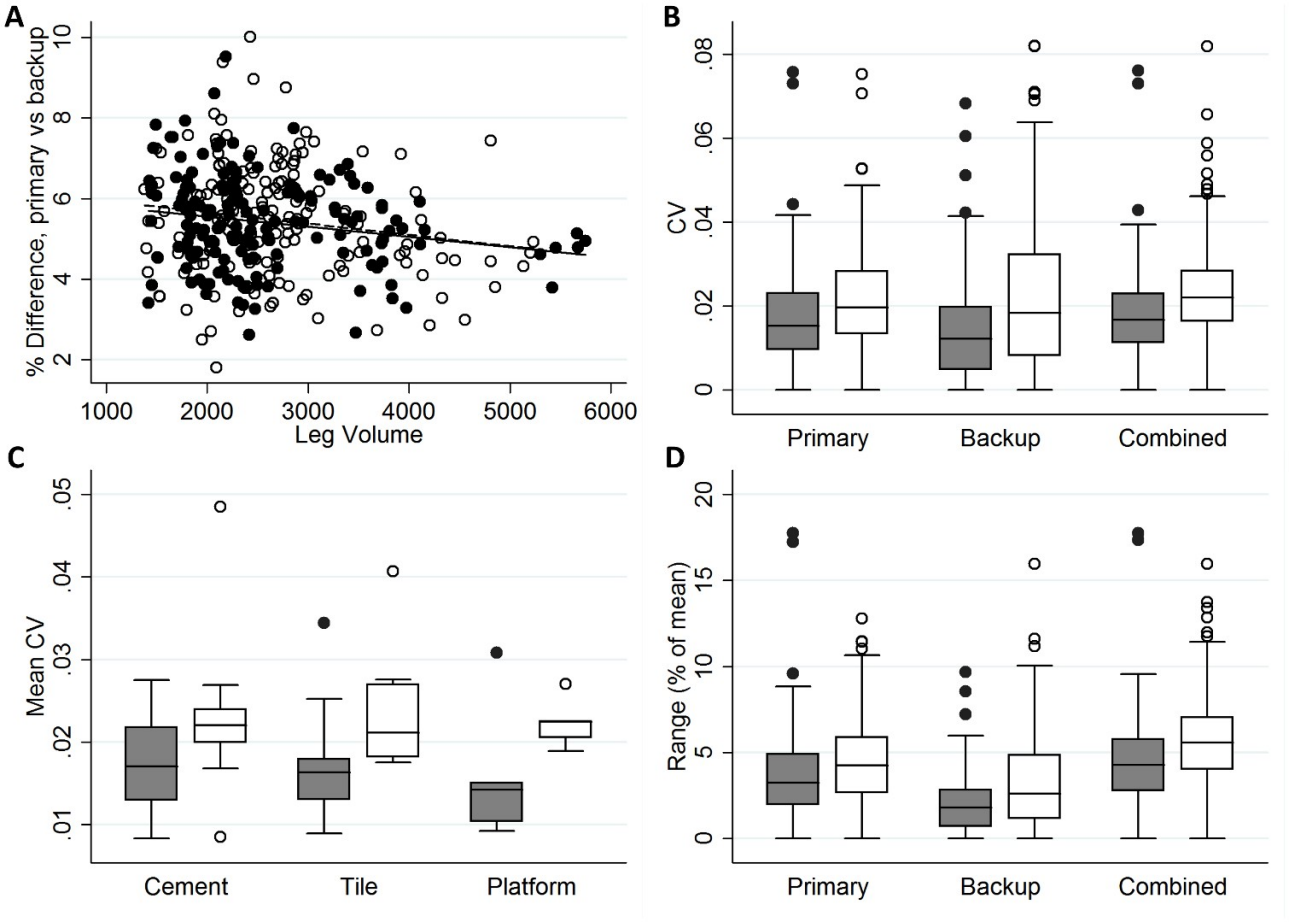
Variation among replicate volume measurements for left leg (gray boxes, closed circles) and right leg measurements (white boxes, open circles). A. Difference between primary and backup volume measurements at visits where both types were done. B. Coefficient of variation (CV) among replicate scans taken using LymphaTech NTD (primary) or LymphaTech v2 (backup) software, or among all scans (primary and backup) after regression to normalize measured volumes (combined). CV was significantly higher (p<0.001) for right leg vs left leg measurements for all comparisons. C. Mean per-participant CV did not vary significantly for right or left leg measurements according to standing surface. D. Range among replicate scans at each visit was also significantly greater (p<0.001) for right leg vs. left leg measurements for all comparisons.

For the majority of visits we acquired four “primary” scans using image-processing software LT-NTD and two “backup” scans using LT-V2. Median coefficient of variation (CV) among replicate primary scan volume measurements performed at the same study visit was 1.5% of the mean (IQR 1.0% - 2.3%) for right legs and 2.0% (IQR 1.3% - 2.8%) for left legs. CV among backup scans was similar: 1.2% (0.5% - 2.0%) for right legs and 1.8% (0.8% - 3.2%) for left legs. Due to a miscalibration in the LT-NTD software that resulted in a mean offset of 5.4% (range 2.0% - 10.0%) between primary and backup volume measurements (Fig 2A), the primary (LT-NTD) measurements were systematically adjusted before examining CV among all (primary and backup) replicate measurements. After adjustment, CV among all replicate volume measurements per visit ranged from 0.0%– 8.2%, which was consistent with the results of our prior study [8]. When analyzed separately, CV for primary scans was not significantly different from CV for backup scans, or from CV for all measures combined. We did not observe any significant differences among intra-visit CVs when stratified by floor type. We were surprised to find, however, that CV was slightly but significantly higher among right leg volume measurements than among left leg volume measurements in all comparisons (Fig 2). The median range (distance between the largest and smallest volumes reported among per-visit replicates) was 4.3% for left leg volumes and 5.6% for right leg volumes; 95% of per-visit ranges for either leg were below 10.8% (Fig 2D **and** S2 Table).

### Intraday volume changes

**Fig 3:**
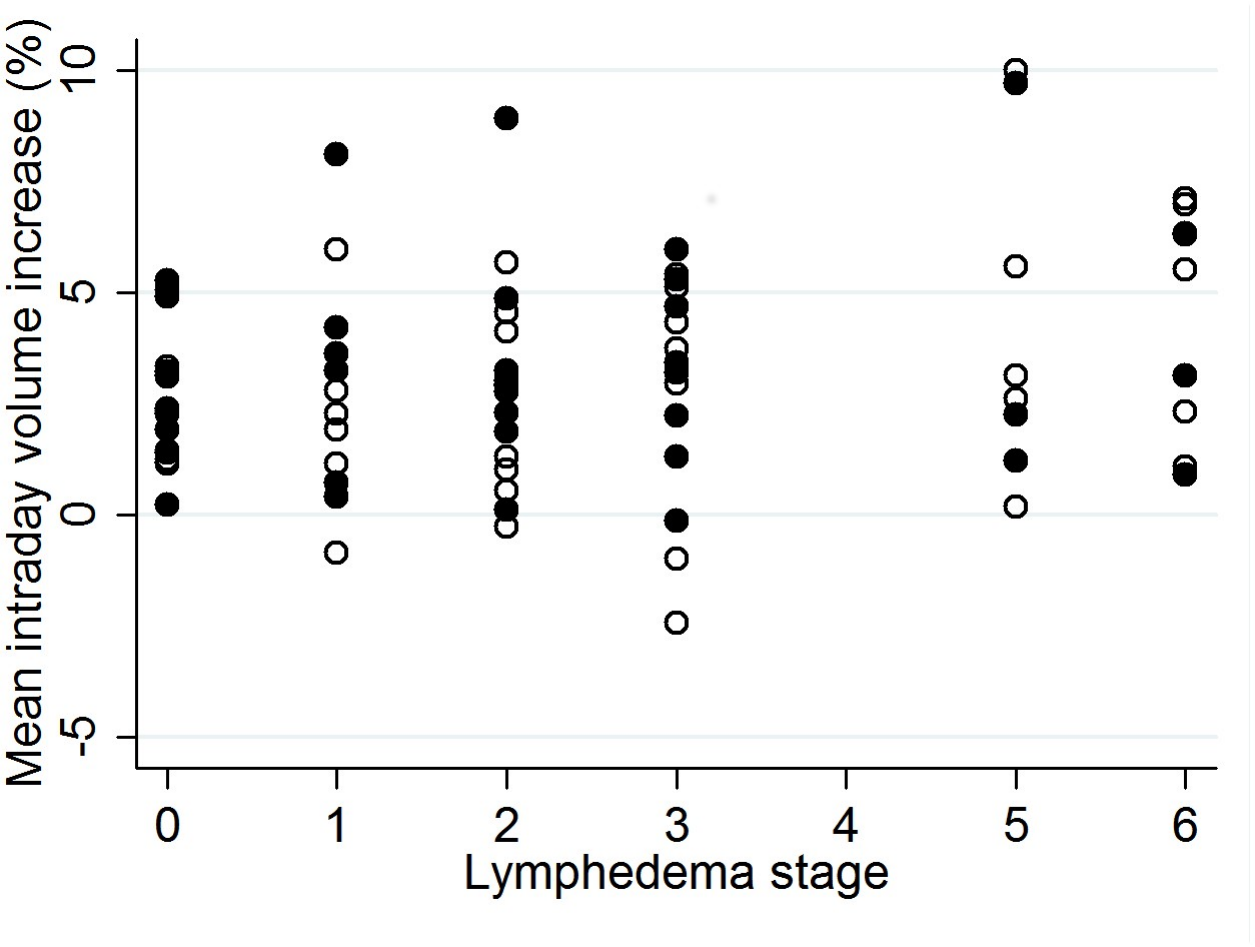
Mean intraday volume increase per leg for left (closed circles) and right (open circles) legs.

The average among replicate measurements for each visit was used to examine visit-to-visit volume changes. Median intra-day increase in limb volumes was 3.1% (range -0.2% - 9.2%) for left legs and 2.8% (range -2.6% - 9.5%) for right legs (Fig 3).

### Day-to-day volume changes

**Fig 4:**
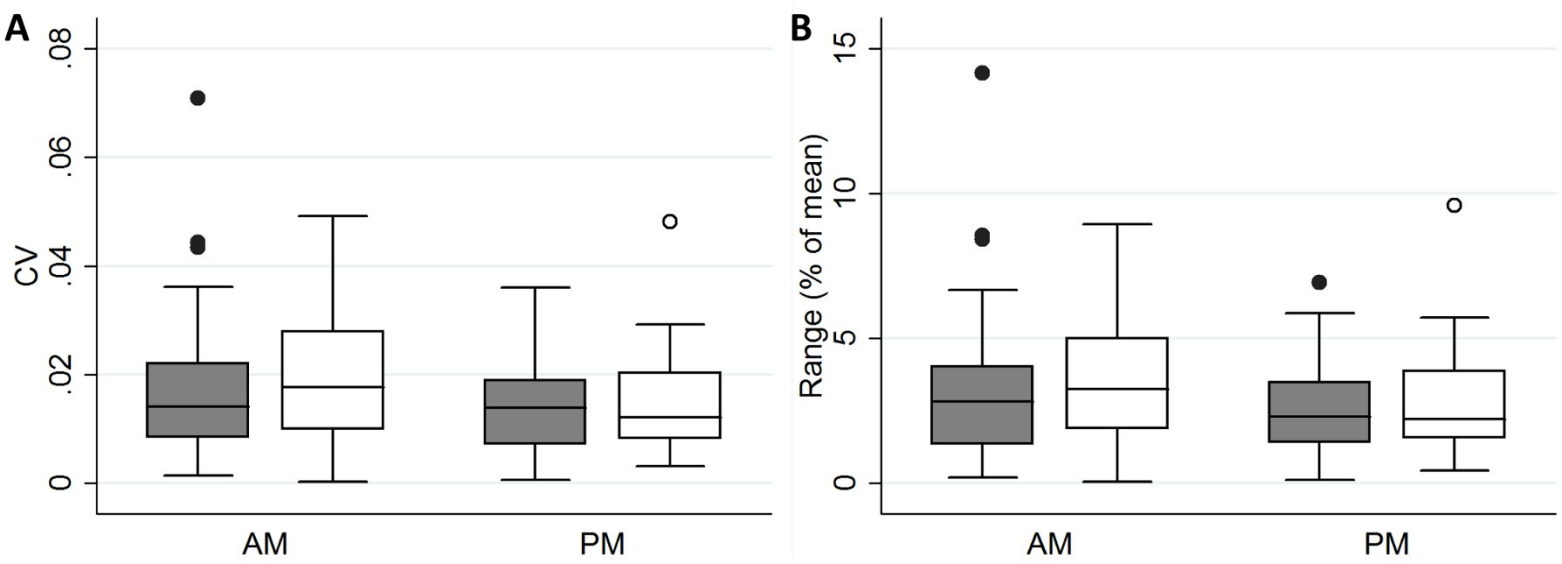
Day-to-day variability in limb volume for left (gray boxes and closed circles) and right (white boxes and open circles) legs. A. Coefficient of variation (CV). B. Range.

To assess how much limb volume changes day-to-day, we compared the per-visit mean volume over the three morning and three afternoon visits for each participant. Day-to-day variability tended to be less for afternoon measurements, but this reached statistical significance only for comparison of CVs among the right leg measurements (p = 0.048) (Fig 4).

### Lymphedema anatomy

**Fig 5:**
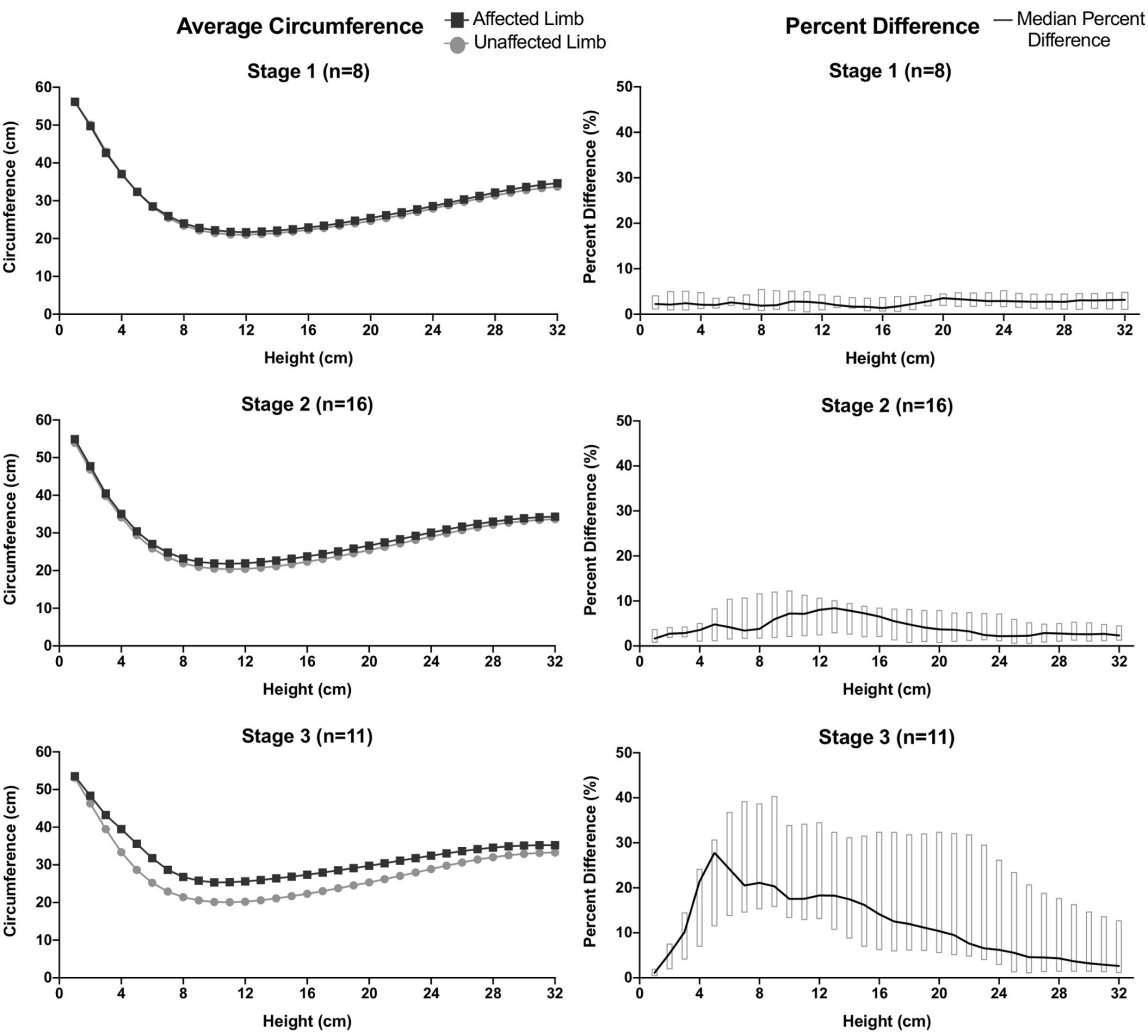
Location of lymphedema among participants with one affected and one unaffected limb. Panels on the left indicate the mean limb circumference of affected limbs (black squares) and unaffected (stage 0) limbs (gray circles) for the group of patients with unilateral lymphedema of the stage indicated. The difference between the circumferences of the affected limb minus the circumferences of the unaffected limb (stage 0) is shown in the right hand panels, Panel titles indicate the number of analyzed limbs in parentheses.

The point cloud model created by 3DIS can be analyzed for any number of circumference or volume measurements. We utilized this advantage to determine where, anatomically, participants with lymphedema are most likely to accumulate excess volume in their affected limbs. Using a subset of point-cloud models from our current (2018) and previous study (2017) [8], we compared circumference measurements between the affected and the unaffected limb for all participants with unilateral stage 1–3 lymphedema (Fig 5). As expected, there was little difference between the affected and unaffected limbs of participants with unilateral stage 1 lymphedema. Among those with unilateral stage 2 lymphedema, the difference in circumference was largest at 12–14 cm of height, just above the narrowest part of the ankle. Those with unilateral stage 3 lymphedema also had a peak circumference difference at 12–14 cm of height, but the largest circumference difference was at 5 cm of height, corresponding to the foot dorsum (Fig 5).

Next, we compared morning and afternoon limb circumference differences to determine where intraday volume increases occur. We suspected volume accumulation would occur primarily in the ankle, but no clear pattern was apparent for any stage (S2 Fig).

Finally, we sought to determine whether 3DIS circumference measurements are prone to greater variability at different heights along the length of the leg. In our prior study, CVs were markedly higher below the ankle (8–12 cm of height) than above the ankle [8]. This discrepancy was greatly reduced in the current study, and this is probably due to improvements in the algorithm for defining the floor/foot border incorporated in the LymphaTech software between these two studies (S3 Fig).

## Discussion

Lymphedema management is a challenge in high and low resource settings and relies heavily on self care to prevent progression of disease and reduce adenolymphangitis episodes [11–13]. Due to the insidious nature of lymphedema progression, tools that allow patients and clinicians to track changes in leg size or appearance over time can be useful indicators of the success (or lack of success) of self care or other efforts to prevent lymphedema progression. Outcomes used to assess the success of hygiene based lymphedema management include frequency of adenolymphangitis episodes, changes in stage, surveys to assess perceived disability/quality of life/depression, and measurements of leg size (i.e. volume or circumference) [14]. Infrared imaging with 3DIS has many advantages over volume displacement and tape measurements of limb circumference as a method for determining limb size. It is non-invasive, faster, and easier for patients [8]. The availability of 3DIS for limb measurements will make it much easier to include anthropometric outcomes in lymphedema research studies and clinical trials, and in clinical care of lymphedema patients. It is therefore important to understand the potential limitations of the technology, how to best implement it, and how to interpret the outcomes.

In our prior study of 3DIS for leg anthropometry among patients with filarial lymphedema, we established the reliability and accuracy of 3DIS in a clinical setting [8]. As a result, 3DIS measurements were added as an outcome measure to ongoing clinical trials in Sri Lanka and elsewhere (ClinicalTrials.gov identifiers: NCT02929134, NCT02927496, NCT02929121). The current study was motivated by questions arising from use of 3DIS in these studies, including: “What best practices should be followed when using 3DIS?”, “What magnitude of volume difference is meaningful when following patients longitudinally?”, and “Does volume need to be measured always at the same time of day to avoid introducing errors due to fluid accumulation throughout the day?” In this study we sought to answer to these questions. Since we wished to visit patients in the morning before they had been on their feet much during the course of the day, this required home visits, which gave us the opportunity to test whether 3DIS measurements are feasible in the home or community.

In the present study, we found that in-home 3DIS measurements are feasible and appear to be as reliable as in-clinic measurements, when done appropriately by trained examiners. Because 3DIS relies on detection of infrared wavelengths, we were concerned that varying reflectivity of different floor types in participant’s homes might affect the reliability of scanner measurements. However, we did not observe any significant differences among intra-visit CVs when stratified by floor type. Furthermore, we found that 3DIS could reliably be done outdoors in the shade if the study team provided a level platform on which patients could stand. These observations indicate that 3DIS can be used to obtain accurate limb volume measurements in remote field settings.

We found that limb volume in our participants increased on average about 3% between morning and evening measurements. This occurred among legs of all stages, including stage 0 (clinically normal limbs). Furthermore, we observed day-to-day volume differences of up to 14% among morning (or evening) scans, although the maximum day-to-day difference was less that 5% for the vast majority of participants. CV among replicate volume measurements in this study was <0.05 for most visits. The range among replicate scans (highest value minus lowest value), which may be a more intuitive quality control measure, was <10% for the vast majority of limbs with a few outliers as high as 18%.

To preserve independence of observations in our analyses, in this study we chose to analyze right and left leg data separately. This led to the surprising finding that volume measurements from replicate scans were less variable for left legs than for right legs. We suspect this may be due our practice of acquiring the scan by circling the participant in a counterclockwise manner, starting in front. This pattern causes the surface of most of the left leg to be captured first, and may make reconstruction of the surface of the right leg more susceptible to artefacts caused by unintentional participant movement. Despite this, we believe operators should use a consistent scanning direction; varying the direction may cause confusion and introduce other types of error.

Another unexpected finding was the trend towards less day-to-day variability for afternoon volumes compared to morning volumes. We anticipated the opposite; that morning volumes would be more consistent because the amount of accumulated volume would be lower. One potential explanation for the trend towards higher consistency in afternoon volumes may be that accumulation of edema occurs early in the day and reaches a plateau by afternoon. In this case our morning visits may have caught participants at different places along the edema “saturation” curve, while the afternoon visits mainly occurred after the majority of the daily volume increase had already occurred.

This study has some important limitations. First, the participant population was small and may not be representative of all patients with filarial lymphedema. In addition, results from patients with filarial lymphedema may not be valid for patients with lymphedema from other causes. Second, we did not attempt to control for the effects of daily activity, such as the amount of time spent standing or sitting, that may have affected daily volume changes. For example, one participant whose leg volumes were smaller in the afternoon than the morning reported to the study team that she spent much of that day in bed. Thus, one should not interpret our data as defining how much intraday volume typically increases in this population, but rather an example of how much it may vary. Third, the unanticipated software problems that prompted our use of two different versions made the estimates of dispersion (CV and range) less robust that they would have been had all measurements been done with the same software. Although we attempted to control for this by systematic adjustment of primary measurements to compensate for the offset in reported volumes, the data would have been more reliable had all measurements been done with the same software.

Ours remain the only publications to date that report on the performance characteristics of the LymphaTech 3DIS system. Our published studies and others in progress have generated a large library of 3D point cloud images that may be useful for secondary studies of lymphedema anatomy. For example, the comparison of affected and unaffected limbs in this study indicates that among patients with stage 2 lymphedema, the peak circumference difference occurs around the ankle at ~13 cm of height. This suggests that if one wished to monitor progression of early stage lymphedema in our population and had only access to tape measurements, doing these measurements at a height of 13 cm would be most likely to reveal differences.

In conducting these studies, we have gained considerable practical experience using the LymphaTech 3DIS. Its strengths include reliability and accuracy that are comparable to water displacement and tape measurements of circumference [8], speed, portability, and convenience for the subjects. Limitations include the initial cost of equipment (a tablet computer plus an infrared sensor, approximately $1,000 US dollars), software licensing costs (LymphaTech has expressed a desire to work with filariasis/podoconiosis clinics and researchers to provide price points suitable for low-resource environments), and the need for adequate training of examiners. As with tape measurements or water displacement, poor technique with 3DIS can result in inaccurate and imprecise measurements. Because the system is easy to use (point the scanner at the person and circle around until the surface of both legs appear white on the scanner screen) examiners sometimes neglect important elements of acquiring scans in the most reproducible way. Errors in technique that we have observed causing flawed scans include poor positioning of the subject (irregular stance, not in the center of the data capture area), movement of the subject during the scan, scanning too quickly or too slowly (poor capture with the former, movement artefact with the latter), scanning in direct sunlight (interference with infrared detection), and failure to carefully review the 3D model for errors at the end of the scan. Each of these errors can be minimized with appropriate training and attention to detail. Based on our experience to date we have generated a list of “Five S’s” for quality scan acquisition that can help ensure data integrity (Table 2).

**Table 2:** Five S’s for quality scan acquisition.

Surface	The subject should stand on a smooth, level, surface that is not in direct sunlight
Stance	The subject should stand straight (no bend in the knees), with both legs perpendicular to the floor, and the feet a standard width apart (use markers on the floor surface or a spacer between the heels)
Sizing box	Adjust the sizing box on the scanner to collect data from the knees and below. Position the box so the subject is in the center of the box, with at least 5 cm of space between the lateral surface of each foot and the box edge.
Speed	A good scan should take 1–2 minutes. Scanning too fast can lead to poor data capture; scanning too slow makes it more likely the subject will move during the scan, which introduces distortions.
Scrutinize	After scanning, carefully review the point-cloud model from all angles. Check the stance and positioning of the subject in the sizing box. Search for signs of movement artefact or poor surface capture. Ensure that no clothing falls over the portion of the leg to be measured. In short, ensure that the 3D model is an accurate representation of the actual surface of the subject’s legs. If it is not, delete the model (do not save the scan for analysis) and start over.

Our findings have some important practical implications for futures studies using 3DIS. First, one should take replicate measurements (at least two) at each visit. This provides more robust volume estimates and also provides important feedback about data quality. Our data suggest one should expect <10% difference between any two replicate measurements (for 95% of our visits, the range among replicates was <8.3% for left legs and <10.8% for right legs). In fact, the range in our data may be artificially inflated due to the high number of replicates. Despite this, 75% of all our visits had a range of <7% among all replicates (using the mean as denominator). Differences >10% between replicates almost certainly indicate that there are errors in one or both of the point cloud models. Therefore, if differences >7% among replicates are frequently noted, investigators should review the 3D models and potentially correct examiner practices. Second, visit-to-visit differences in volume may be as high as 5–7% simply due to day-to-day variability. Finally, day-to-day variability in limb volumes trended lower for afternoon measurements in our study, but the magnitude of this difference was small. It may not matter, therefore, whether longitudinal measurements are taken in the morning or the afternoon, but taking longitudinal measurements at the same time of day whenever possible should reduce variability caused by intraday volume accumulation.

## Supporting information

S1 Table Number of scans taken at each visit and number of scans included in final analysis. (DOCX)

S2 Table Coefficient of variation among replicate scans at each visit. (DOCX)

S1 Fig Point cloud models excluded from analysis due to artefact. Images are ordered as shown in the S1 Figure file, moving across rows from left to right first, then moving to the next row.
(PDF)

S2 Fig Percent differences in PM—AM circumference measurements of participant limbs, by stage. Number of analyzed limbs are indicated in the panel title in parentheses. Bars represent range of observed values.
(PDF)

S3 Fig Circumference measurements and CV among circumference measurements among limb scans in our prior study (2017) [8] and in the current study (2018). Number of analyzed scans are indicated in the legend in parentheses.
(PDF)
