# Supplementary material for: Portable infrared imaging for longitudinal limb volume monitoring in patients with lymphatic filariasis
Source: PLoS Negl Trop Dis. 2019 Oct 4;13(10):e0007762. doi: 10.1371/journal.pntd.0007762 (PMC6795459; doi:10.1371/journal.pntd.0007762)
Supplement: S1 Table — (DOCX) [file pntd.0007762.s001.docx]

| **S1 Table. Number of scans taken at each visit and number of scans included in final analysis** | | | | | | | | | | | | | | | |
| --- | --- | --- | --- | --- | --- | --- | --- | --- | --- | --- | --- | --- | --- | --- | --- |
| **Total scans** | | | | | | | | **Retained scans** | | | | | | | |
| **Participant ID** | **AM1** | **AM2** | **AM3** | **PM1** | **PM2** | **PM3** | **Total** | **Participant ID** | **AM1** | **AM2** | **AM3** | **PM1** | **PM2** | **PM3** | **Total** |
| 20 | 6 | 6 | 6 | 6 | 6 | 6 | 36 | 20 | 4 | 6 | 5 | 4 | 6 | 6 | 31 |
| 21 | 0 | 4 | 4 | 4 | 4 | 4 | 20 | 21 | 0 | 4 | 4 | 4 | 4 | 4 | 20 |
| 22 | 6 | 6 | 6 | 6 | 6 | 6 | 36 | 22 | 6 | 6 | 5 | 6 | 5 | 6 | 34 |
| 23 | 6 | 6 | 6 | 6 | 6 | 6 | 36 | 23 | 5 | 6 | 4 | 6 | 6 | 4 | 31 |
| 24 | 6 | 6 | 0 | 6 | 6 | 0 | 24 | 24 | 6 | 6 | 0 | 6 | 6 | 0 | 24 |
| 25 | 6 | 6 | 6 | 6 | 6 | 6 | 36 | 25 | 6 | 6 | 6 | 6 | 6 | 6 | 36 |
| 26 | 7 | 6 | 6 | 6 | 6 | 6 | 37 | 26 | 6 | 6 | 6 | 6 | 6 | 6 | 36 |
| 27 | 6 | 5 | 6 | 6 | 6 | 6 | 35 | 27 | 4 | 5 | 6 | 3 | 5 | 6 | 29 |
| 28 | 6 | 6 | 6 | 6 | 6 | 6 | 36 | 28 | 5 | 6 | 6 | 6 | 6 | 6 | 35 |
| 29 | 6 | 6 | 6 | 6 | 6 | 6 | 36 | 29 | 6 | 6 | 6 | 6 | 6 | 6 | 36 |
| 30 | 6 | 6 | 6 | 6 | 6 | 6 | 36 | 30 | 6 | 6 | 6 | 5 | 5 | 5 | 33 |
| 31 | 4 | 4 | 4 | 4 | 4 | 4 | 24 | 31 | 2 | 3 | 3 | 3 | 4 | 4 | 19 |
| 32 | 6 | 6 | 7 | 6 | 6 | 6 | 37 | 32 | 6 | 5 | 7 | 6 | 6 | 6 | 36 |
| 33 | 6 | 6 | 6 | 6 | 6 | 6 | 36 | 33 | 6 | 6 | 6 | 6 | 6 | 6 | 36 |
| 34 | 6 | 6 | 6 | 6 | 6 | 6 | 36 | 34 | 6 | 6 | 6 | 6 | 6 | 6 | 36 |
| 35 | 6 | 6 | 6 | 6 | 6 | 5 | 35 | 35 | 6 | 5 | 6 | 5 | 5 | 5 | 32 |
| 36 | 6 | 6 | 6 | 6 | 6 | 6 | 36 | 36 | 6 | 6 | 6 | 6 | 6 | 5 | 35 |
| 37 | 6 | 6 | 6 | 6 | 6 | 6 | 36 | 37 | 6 | 6 | 6 | 6 | 6 | 6 | 36 |
| 38 | 6 | 6 | 6 | 6 | 6 | 6 | 36 | 38 | 6 | 6 | 6 | 6 | 6 | 6 | 36 |
| 39 | 6 | 6 | 6 | 6 | 6 | 6 | 36 | 39 | 6 | 6 | 6 | 6 | 6 | 6 | 36 |
| 40 | 6 | 7 | 6 | 6 | 6 | 6 | 37 | 40 | 6 | 7 | 6 | 6 | 5 | 6 | 36 |
| 41 | 6 | 6 | 6 | 6 | 6 | 6 | 36 | 41 | 6 | 6 | 6 | 6 | 6 | 6 | 36 |
| 42 | 6 | 6 | 6 | 6 | 6 | 6 | 36 | 42 | 6 | 6 | 6 | 6 | 6 | 6 | 36 |
| 43 | 6 | 6 | 6 | 6 | 6 | 6 | 36 | 43 | 6 | 6 | 6 | 5 | 6 | 6 | 35 |
| 44 | 6 | 6 | 6 | 5 | 6 | 6 | 35 | 44 | 6 | 6 | 6 | 5 | 6 | 6 | 35 |
| 45 | 6 | 6 | 6 | 5 | 6 | 6 | 35 | 45 | 6 | 6 | 6 | 5 | 6 | 6 | 35 |
| 46 | 4 | 6 | 4 | 3 | 4 | 4 | 25 | 46 | 3 | 4 | 3 | 1 | 4 | 4 | 19 |
| 47 | 7 | 6 | 6 | 4 | 6 | 6 | 35 | 47 | 7 | 6 | 5 | 4 | 6 | 6 | 34 |
| 48 | 3 | 6 | 6 | 6 | 6 | 6 | 33 | 48 | 3 | 6 | 6 | 6 | 6 | 6 | 33 |
| 49 | 0 | 6 | 6 | 0 | 6 | 6 | 24 | 49 | 0 | 6 | 6 | 0 | 6 | 6 | 24 |
| 50 | 0 | 5 | 6 | 0 | 6 | 6 | 23 | 50 | 0 | 5 | 6 | 0 | 6 | 6 | 23 |
| 51 | 0 | 6 | 6 | 0 | 6 | 6 | 24 | 51 | 0 | 6 | 6 | 0 | 6 | 6 | 24 |
| 52 | 0 | 6 | 6 | 0 | 6 | 6 | 24 | 52 | 0 | 6 | 6 | 0 | 6 | 6 | 24 |
| 53 | 0 | 6 | 6 | 0 | 6 | 6 | 24 | 53 | 0 | 6 | 6 | 0 | 6 | 6 | 24 |
| 54 | 0 | 6 | 6 | 0 | 6 | 6 | 24 | 54 | 0 | 6 | 6 | 0 | 6 | 6 | 24 |
| 55 | 2 | 3 | 2 | 2 | 2 | 2 | 13 | 55 | 2 | 3 | 2 | 2 | 2 | 2 | 13 |
| 56 | 4 | 2 | 3 | 2 | 2 | 2 | 15 | 56 | 4 | 2 | 3 | 2 | 2 | 2 | 15 |
| 57 | 4 | 4 | 4 | 4 | 4 | 4 | 24 | 57 | 4 | 4 | 4 | 4 | 4 | 4 | 24 |
| 58 | 5 | 4 | 4 | 4 | 4 | 4 | 25 | 58 | 4 | 4 | 4 | 4 | 4 | 4 | 24 |
| 59 | 4 | 4 | 4 | 4 | 4 | 4 | 24 | 59 | 4 | 4 | 4 | 4 | 4 | 4 | 24 |
| 60 | 4 | 4 | 4 | 4 | 4 | 4 | 24 | 60 | 4 | 4 | 4 | 4 | 4 | 4 | 24 |
| **Total** | **186** | **226** | **220** | **183** | **224** | **217** | **1256** | **Total** | **175** | **221** | **213** | **172** | **219** | **213** | **1,213** |
