# Supplementary material for: Portable infrared imaging for longitudinal limb volume monitoring in patients with lymphatic filariasis
Source: PLoS Negl Trop Dis. 2019 Oct 4;13(10):e0007762. doi: 10.1371/journal.pntd.0007762 (PMC6795459; doi:10.1371/journal.pntd.0007762)
Supplement: S2 Table — (DOCX) [file pntd.0007762.s002.docx]

| **S2 Table. Coefficient of variation among replicate scans at each visit** | | | |  |
| --- | --- | --- | --- | --- |
| **Scan type (leg)** | **N visits** | **Scans per visit*** | **CV† (IQR)** | **Range** |
| Primary (Right) | 224 | 4 | 1.5% (1.0% - 2.3%) | 0.0% - 7.6% |
| Primary (Left) | 224 | 4 | 2.0% (1.3% - 2.8%) | 0.0% - 7.5% |
| Backup (Right) | 170 | 2 | 1.2% (0.5% - 2.0%) | 0.0% - 6.8% |
| Backup (Left) | 170 | 2 | 1.8% (0.8% - 3.2%) | 0.0% - 8.2% |
| Combined (Left) | 230 | 6 | 1.7% (1.1% - 2.3%) | 0.0% - 7.6% |
| Combined (Right) | 230 | 6 | 2.2% (1.6% - 2.8%) | 0.0% - 8.2% |
| * There were 20 visits with only two primary scans and 20 visits with only 3 primary scans analyzed. There were four visits with 3 backup scans and nine visits with 4 backup scans analyzed.  †Expressed as percentage of the mean | | | | |
