# Supplementary material for: Portable infrared imaging for longitudinal limb volume monitoring in patients with lymphatic filariasis
Source: PLoS Negl Trop Dis. 2019 Oct 4;13(10):e0007762. doi: 10.1371/journal.pntd.0007762 (PMC6795459; doi:10.1371/journal.pntd.0007762)
Supplement: S1 Fig — Images are ordered as shown in the S1 Figure file, moving across rows from left to right first, then moving to the next row. (PDF) [file pntd.0007762.s003.pdf]

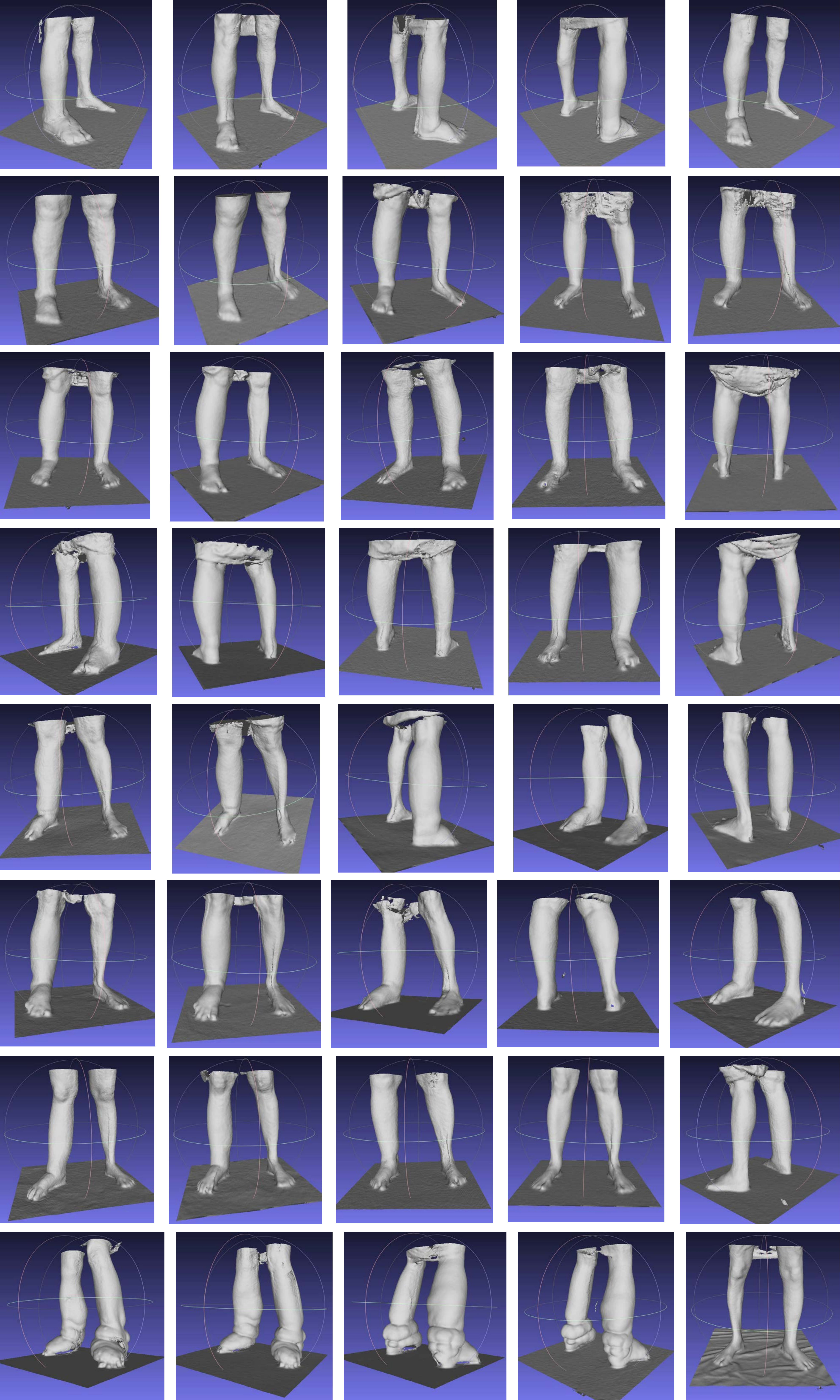

Participant	Day	Visit	Reason for Exclusion	Notes
20	1	AM	movement artefact	multiple artefacts
20	1	AM	movement artefact	multiple artefacts, including Swiss roll right anterior ankle
20	1	PM	movement artefact	poor capture right foot
20	1	PM	movement artefact	bad Swiss roll right posterior ankle
20	3	AM	movement artefact	right anterior ankle Swiss roll
22	2	PM	movement artefact	left medial ankle cut
22	3	AM	movement artefact	left medial ankle cut
23	1	AM	movement artefact	left medial ankle cut
23	3	AM	movement artefact	ankle cuts both legs
23	3	AM	movement artefact	severe left medial ankle cut
23	3	PM	movement artefact	left medial foot and anterior ankle artefact
23	3	PM	movement artefact	left medial ankle and heel artefacts
26	1	AM	poor surface capture	fuzzy appearance of leg surfaces
27	1	AM	poor surface capture	fuzzy surfaces; also has some movement artefact
27	1	AM	movement artefact	right posterior ankle Swiss roll
27	1	PM	movement artefact	striping and severe movement artefacts left medial and right anterior ankles
27	1	PM	movement artefact	right posterior ankle Swiss roll
27	1	PM	movement artefact	Swiss rolls both ankles, divot in right heel
27	2	PM	movement artefact	Swiss roll right anterior ankle; poor capture right great toe
28	1	AM	movement artefact	severe cut left medial ankle; Swiss roll right posterior ankle
30	1	PM	movement artefact	cut left medial foot and anteromedial ankle
30	2	PM	movement artefact	left anterior shin cut
30	3	PM	movement artefact	left posteromedial foot and ankle Swiss roll
31	1	AM	movement artefact	left anterolateral ankle and right medial knee Swiss rolls
31	1	AM	movement artefact	Swiss rolls both posterior feet and ankles
31	1	PM	movement artefact	cut left medial foot and ankle
31	2	AM	movement artefact	Swiss roll left anterior shin and ankle
31	3	AM	movement artefact	left anterior ankle divot
32	2	AM	floater	floater medial to left ankle; small area not captured right heel
35	1	PM	floater	floaters posterior to left ankle
35	2	AM	movement artefact	left anteromedial shin Swiss roll
35	2	PM	movement artefact	both medial feet
36	3	PM	movement artefact	left anterior shin Swiss roll
40	2	PM	movement artefact	left anterior shin Swiss roll
43	1	PM	floater	floater posterior to left heel
46	1	AM	poor surface capture	poor capture around deep folds both feet and ankles
46	1	PM	movement artefact	Swiss roll left lateral leg
46	1	PM	poor surface capture	poor capture under deep folds both feet; also Swiss roll artefact left medal leg
46	2	AM	poor surface capture	poor capture under deep folds both feet (photo omitted)
46	2	AM	floater	floater posterior to right leg; also Swiss left medal leg
46	3	AM	movement artefact	marked Swiss rolls left anterior ankle and foot (photo omitted)
58	1	AM	inappropriate standing surface	standing on a sheet
