# Supplementary material for: Portable infrared imaging for longitudinal limb volume monitoring in patients with lymphatic filariasis
Source: PLoS Negl Trop Dis. 2019 Oct 4;13(10):e0007762. doi: 10.1371/journal.pntd.0007762 (PMC6795459; doi:10.1371/journal.pntd.0007762)
Supplement: S2 Fig — Number of analyzed limbs are indicated in the panel title in parentheses. Bars represent range of observed values. (PDF) [file pntd.0007762.s004.pdf]

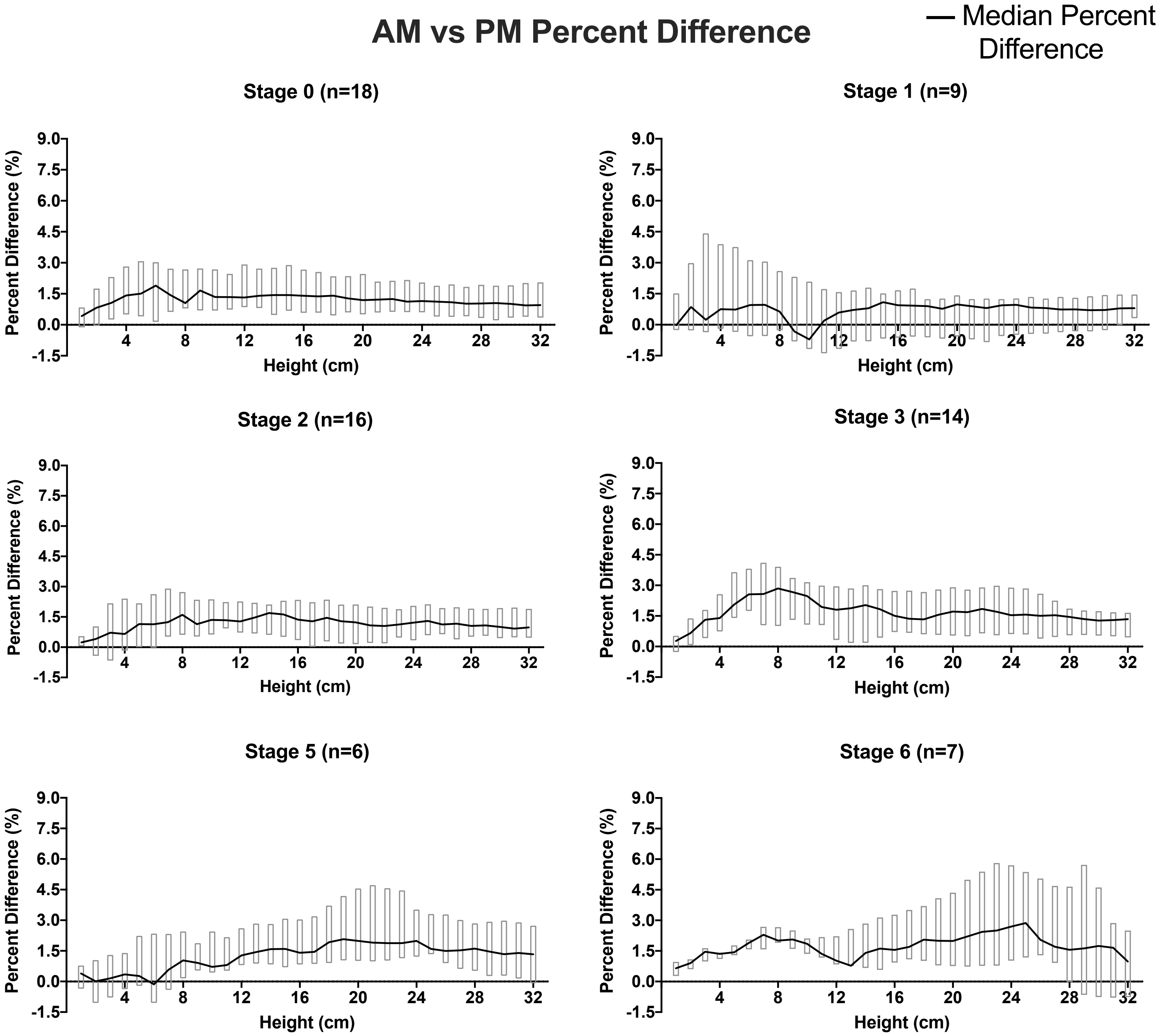
AM vs PM Percent Difference
Median Percent Difference
Stage 0 (n=18)
Stage 1 (n=9)
Stage 2 (n=16)
Stage 3 (n=14)
Stage 5 (n=6)
Stage 6 (n=7)
Percent Difference (%)
Height (cm)
9.0
7.5
6.0
4.5
3.0
1.5
0.0
-1.5
4
8
12
16
20
24
28
32
