# Supplementary material for: Portable infrared imaging for longitudinal limb volume monitoring in patients with lymphatic filariasis
Source: PLoS Negl Trop Dis. 2019 Oct 4;13(10):e0007762. doi: 10.1371/journal.pntd.0007762 (PMC6795459; doi:10.1371/journal.pntd.0007762)
Supplement: S3 Fig — Number of analyzed scans are indicated in the legend in parentheses. (PDF) [file pntd.0007762.s005.pdf]

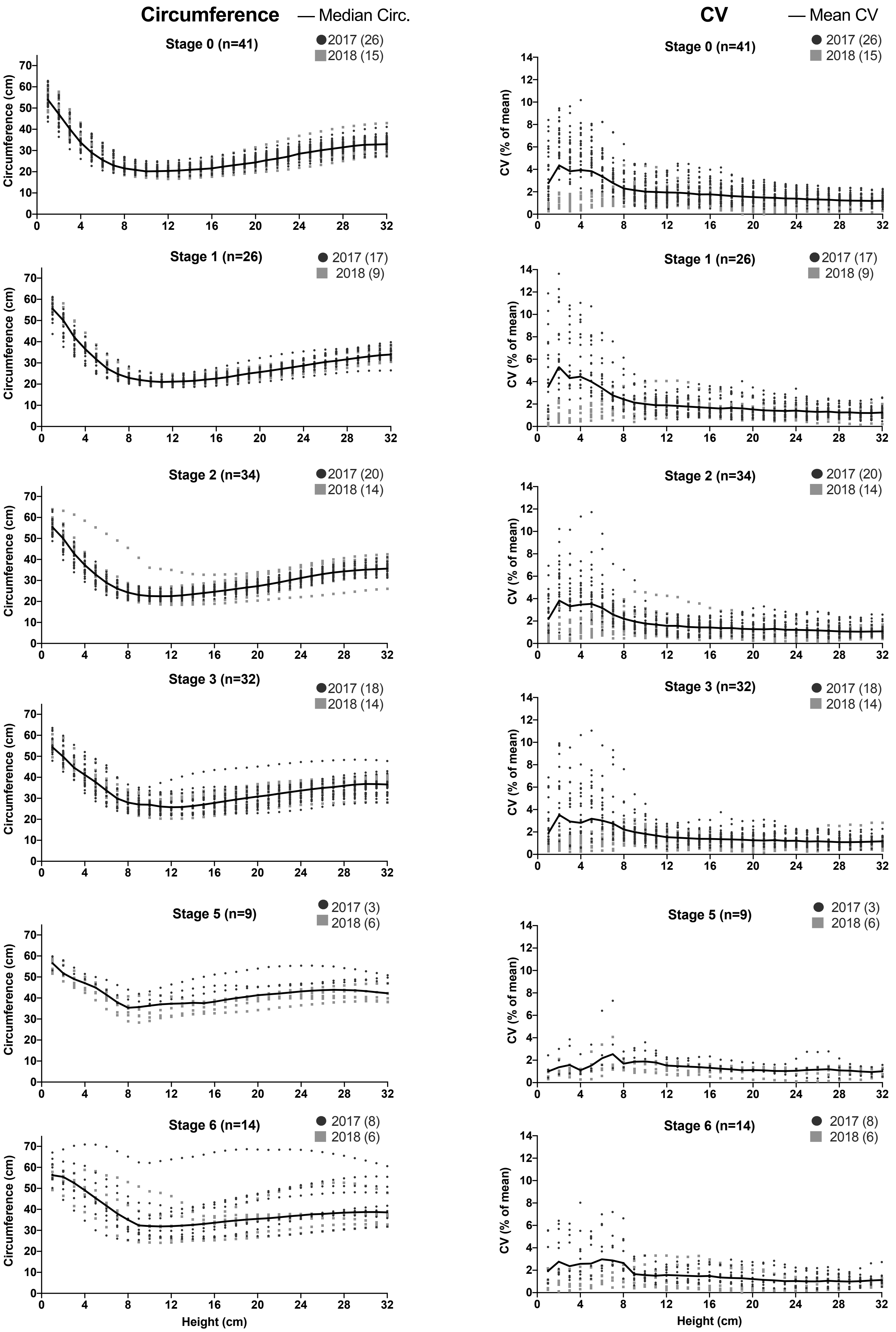
Circumference
— Median Circ.
CV
— Mean CV
Stage 0 (n=41)
2017 (26)
2018 (15)
Stage 1 (n=26)
2017 (17)
2018 (9)
Stage 2 (n=34)
2017 (20)
2018 (14)
Stage 3 (n=32)
2017 (18)
2018 (14)
Stage 5 (n=9)
2017 (3)
2018 (6)
Stage 6 (n=14)
2017 (8)
2018 (6)
Circumference (cm)
CV (% of mean)
Height (cm)
